# The signed two-space proximity model for learning representations in protein–protein interaction networks

**DOI:** 10.1093/bioinformatics/btaf204

**Published:** 2025-04-23

**Authors:** Nikolaos Nakis, Chrysoula Kosma, Anastasia Brativnyk, Michail Chatzianastasis, Iakovos Evdaimon, Michalis Vazirgiannis

**Affiliations:** École Polytechnique, LIX, Institute Polytechnique de Paris, Palaiseau, 91120, France; ENS Paris Saclay, CNRS, SSA INSERM, Université Paris-Saclay, Université Paris Cité, 91190, Gif-sur-Yvette, France; Ancient Genomics Laboratory, The Francis Crick Institute, London, NW1 1AT, United Kingdom; École Polytechnique, LIX, Institute Polytechnique de Paris, Palaiseau, 91120, France; École Polytechnique, LIX, Institute Polytechnique de Paris, Palaiseau, 91120, France; École Polytechnique, LIX, Institute Polytechnique de Paris, Palaiseau, 91120, France; MBZUAI, Mohamed bin Zayed, University of Artificial Intelligence, Abu Dhabi, United Arab Emirates

## Abstract

**Motivation:**

Accurately predicting complex protein–protein interactions (PPIs) is crucial for decoding biological processes, from cellular functioning to disease mechanisms. However, experimental methods for determining PPIs are computationally expensive. Thus, attention has been recently drawn to machine learning approaches. Furthermore, insufficient effort has been made toward analyzing signed PPI networks, which capture both activating (positive) and inhibitory (negative) interactions. To accurately represent biological relationships, we present the Signed Two-Space Proximity Model (S2-SPM) for signed PPI networks, which explicitly incorporates both types of interactions, reflecting the complex regulatory mechanisms within biological systems. This is achieved by leveraging two independent latent spaces to differentiate between positive and negative interactions while representing protein similarity through proximity in these spaces. Our approach also enables the identification of archetypes representing extreme protein profiles.

**Results:**

S2-SPM’s superior performance in predicting the presence and sign of interactions in SPPI networks is demonstrated in link prediction tasks against relevant baseline methods. Additionally, the biological prevalence of the identified archetypes is confirmed by an enrichment analysis of Gene Ontology (GO) terms, which reveals that distinct biological tasks are associated with archetypal groups formed by both interactions. This study is also validated regarding statistical significance and sensitivity analysis, providing insights into the functional roles of different interaction types. Finally, the robustness and consistency of the extracted archetype structures are confirmed using the Bayesian Normalized Mutual Information (BNMI) metric, proving the model’s reliability in capturing meaningful SPPI patterns.

**Availability:**

S2-SPM is implemented and freely available under the MIT license at https://github.com/Nicknakis/S2SPM.

## 1 Introduction

Proteins interact with each other to carry out various cellular functions (Legrain *et al.* 2001), forming complex networks within biological pathways known as the interactome (Cusick *et al.* 2005). Capturing protein–protein interactions (PPIs) is fundamental for decoding cellular processes, crucially implicated in disease mechanisms (Rual *et al.* 2005). However, experimental methods for the determination of PPIs, such as yeast two-hybrid systems (Ito *et al.* 2001) and mass spectrometry (Gavin *et al.* 2002), are costly, time-consuming, and insufficient (Han *et al.* 2005). Recently, machine learning (ML) techniques have provided accurate alternatives to overcome these challenges.

ML methods for modeling PPI networks (Soleymani *et al.* 2022; Tang *et al.* 2023) fall into two main categories: (i) sequence- and structure-based approaches that extract protein representations directly from the primary sequence or 3D structure of a protein, including features such as amino acid composition, motifs, and structural domains and (ii) link prediction methods that treat the PPI network as a graph, using its topology to extract network embeddings and interactions. Sequence-based methods range from traditional methods such as SVMs (Guo *et al.* 2010) to neural networks (Zeng *et al.* 2020) that capture nonlinear relationships. Graph-based approaches, built on graph neural networks (GNNs) (Fout *et al.* 2017; Tang *et al.* 2024), integrate sequence and structural information for improved predictions. Specifically, structure-based methods utilize 3D protein data (Liu *et al.* 2020), with recent advancements incorporating hierarchical GNN architectures (Gao *et al.* 2023). On the other hand, link prediction techniques, such as L3 (Kovács *et al.* 2019) and similarity-based methods (Yuen and Jansson 2023), focus on network topology, considering properties such as node degrees and community partitions.

Protein–protein interaction networks can be modeled as signed, capturing both activating (positive) and inhibitory (negative) interactions, extending prediction beyond the presence of a link. This enables a more natural representation of biological relationships, essential for modeling complex regulatory mechanisms (Pritykin and Singh 2013). Several representation methods for signed graphs have been proposed over the years for tasks spanning from community detection (Esmailian and Jalili 2015) to link prediction (Garg *et al.* 2013), mostly for social network applications. Representative models have been built upon the psychologically based concept of balance theory (Cartwright and Harary 1956) and random walks (Perozzi *et al.* 2014) to capture interactions, such as SIDE (Kim *et al.* 2018) and POLE (Huang *et al.* 2022). Extending these ideas with neural networks, SiGNET (Islam *et al.* 2018) combines multi-layer perceptrons with balance theory, and SLF (Xu *et al.* 2019) introduces multiple latent factors that model additional types of interactions. Built upon GNNs, SiGAT (Huang *et al.* 2019) combines common signed network concepts with graph attention networks. A more recent approach, SPMF (Xu *et al.* 2023), extracts node representations using low-rank matrix approximation to better encode multi-order signed proximity.

Unsupervised learning and clustering techniques play a key role in uncovering hidden patterns in protein–protein networks (Pizzuti and Rombo 2014). Archetypal Analysis (AA) (Cutler and Breiman 1994; Mørup and Hansen 2010), originally developed for analyzing observational data within a *K*-dimensional polytope, has been extended to fields like computer vision (Chen *et al.* 2014) and population genetics (Gimbernat-Mayol *et al.* 2021). More recently, AA has been adapted for relational data (Nakis *et al.* 2023, 2024), including applications to signed social networks under the Skellam distribution (Skellam, 1946). The SLIM method (Nakis *et al.* 2023) introduces a unified embedding space, where positive links bring nodes closer in a latent “sociotope,” while negative links push them apart. Although effective for modeling social relationships, this approach does not directly extend to signed protein–protein interaction networks (SPPI) and requires additional modeling adaptations.

In this study, we propose the Signed Two Space Proximity Model (S2-SPM), the first archetypal-based signed network specifically tailored to model protein interactions. The proposed method contributes to the existing research in the field in the following key aspects: (i) S2-SPM outperforms all compared baselines in terms of the tasks of sign and signed link prediction across three real-world PPI networks by 4.3% on average in F1 score with regards to the best competitor, (ii) S2-SPM is supported by an enrichment analysis based on the Gene Ontology (GO) terms, clarifying the biological relevance of the identified archetypes. This analysis statistically confirms the validity of the obtained representations and enables potential explainability aspects that can be crucial for the biomedical research community, (iii) Extensive visualizations of the obtained latent structures and archetypes highlight the effectiveness of the proposed S2-SPM in discovering and capturing latent structures present in SPPI networks, (iv) The consistency and robustness of the extracted structures and archetypes are confirmed through informative measures, such as the Bayesian Normalized Mutual Information score.

## 2 Materials and methods

### 2.1 Archetypal analysis

Archetypes are regarded as the extreme points of the convex hull encompassing the data. Specifically, archetypes refer to the most representative or extreme examples within the dataset, which can be used to understand the essential characteristics or patterns present in the data. They serve as the utmost manifestations of data traits and profiles and can essentially be used to express the data structure in terms of underlying “archetypal” patterns while facilitating the identification and interpretation of such traits.

Formally, for a given data matrix $X\in R^{P\times N}$ such as $X=\{x_{1},x_{2},\ldots ,x_{n}\}$, we aim to extract the archetype matrix $A\in R^{P\times K}$, $A=\{a_{1},a_{2},\ldots ,a_{K}\}$ where K≪P such that:

(1) $$\alpha_{j}=\sum_{i=1}^{N}x_{i}c_{ij},$$

with $c_{n}\in \Delta^{N-1}$, where $\Delta^{N-1}$ denotes the standard simplex in *N* dimensions such that $c_{i}\geq 0$ and $||c|{|}_{1}=1$, (i.e., $\sum_{i}c_{i}=1$). Given that A is also the convex hull of the data, each point $x_{i}$ can now be reconstructed as:

(2) $$x_{i}=\sum_{j=1}^{K}a_{j}z_{ji}$$

where $z_{k}\in \Delta^{K-1}$, $\Delta^{K-1}$ denotes the *K*-dimensional simplex. The matrix Z essentially describes how each data point is expressed as the convex combination of the archetypes defined by A. The previous can be summarized in a matrix form as:

(3) $$X\approx \mathrm{XCZ}\;s.t.\;c_{n}\in \Delta^{N-1}\;\text{and}\;z_{k}\in \Delta^{K-1}.$$

The archetypes in this formulation are represented by the columns of A=XC, defining the corners of the convex hull, represented as the convex combinations of the data.

### 2.1.1 Archetypal analysis for signed protein–protein interaction networks

In SPPI networks (see Definition 1), positive and negative interactions should explicitly be accounted for by their proximity in the latent space. Specifically, we study SPPI networks where protein interactions take a positive or a negative sign based on the regulation effect (up-regulation/down-regulation). Thus, it is important to analyze and visualize both types of relations, defining nodes as similar and positioning them in close proximity in the latent space. This differentiates from the simplistic assumption in social networks (Nakis *et al.* 2023) that dissimilar nodes usually interact negatively. Indeed, from a biological point of view, a negative protein–protein interaction should not be translated as animosity between the proteins, as in the case of a “dislike” in social networks. Thus, in this study, we aim to analyze SPPI networks by differentiating between the signs of interactions in terms of modeling and defining protein similarity independently of the sign of interaction.

Definition 1. *A signed protein–protein interaction network (SPPI) is a biological network in which nodes represent proteins and edges represent regulatory interactions between them. Each edge is annotated with a sign to specify the regulatory outcome of the interaction: a* ***positive***  (+)  *edge indicates an up-regulatory effect, where the interaction leads to increased activity or expression of the target protein, while a* ***negative***  (−)  *edge signifies a down-regulatory effect, where the interaction results in suppression or inhibition of the target protein.*

#### 2.1.2 The signed two-space proximity model (S2-SPM)

We next aim to propose a framework for analyzing SPPI networks by projecting them into two independent latent spaces. One space models positive interactions through close proximity, while the other captures negative interactions in a similar fashion. Each latent space is further designed to facilitate archetypal analysis and the characterization of extreme protein profiles within its respective interaction type. This dual-space approach extends previous work (Nakis *et al.* 2023)—which used a single latent space for social network analysis—by more accurately representing the distinct relational structures inherent to positive and negative interactions. Formally, we aim to learn two sets of latent node representations ${\left\{z_{i}\right\}}_{i\in V}\in R^{K}$, and ${\left\{w_{i}\right\}}_{i\in V}\in R^{K}$, defining the two low-dimensional spaces for a given signed network G=(V,Y) (K≪|V|). We assume that the edges of the signed graph can take any integer value representing the intensity of the interaction and the sign (positive/negative) representing the type of connection (up-regulation/down-regulation) of the protein pair. We utilize the Skellam distribution, which is the difference of two independent Poisson-distributed random variables ($y=N_{1}-N_{2}\in Z$) with respect to the rates $\lambda^{+}$ and $\lambda^{-}$:

$$P(y|\lambda^{+},\lambda^{-})=e^{-(\lambda^{+}+\lambda^{-})}{\left(\frac{\lambda^{+}}{\lambda^{-}}\right)}^{y/2}I_{\left|y\right|}\left(2\sqrt{\lambda^{+}\lambda^{-}}\right),$$

where $N_{1}∼\mathrm{Pois}(\lambda^{+})$ and $N_{2}∼\mathrm{Pois}(\lambda^{-})$, and $I_{\left|y\right|}$ is the modified Bessel function of the first kind and order |y|. In general, $\lambda^{+}$ generates the intensity of a positive outcome for *y* while $\lambda^{-}$ that of a negative outcome. Consequently, we can obtain the negative log-likelihood, which acts as our loss function, as:

$$\begin{array}{c} L(Y):=-\text{log}\;p(y_{ij}|\lambda_{ij}^{+},\lambda_{ij}^{-})\\ =\sum_{i<j}\left(\lambda_{ij}^{+}+\lambda_{ij}^{-}\right)-\frac{y_{ij}}{2}\text{log}\;\left(\frac{\lambda_{ij}^{+}}{\lambda_{ij}^{-}}\right)-\text{log}(I_{ij}^{*}), \end{array}$$

where $I_{ij}^{*}:=I_{\left|y_{ij}\right|}\left(2\sqrt{\lambda_{ij}^{+}\lambda_{ij}^{-}}\right)$. We, here, do not assume any priors over the parameters of the model, contrary to Nakis *et al.* (2023), as we observed better model performance in the former case. Assuming relational data as input, the Skellam distribution rate parameter $\lambda_{ij}^{+}$ is responsible for modeling the intensity of a positive interaction, whereas $\lambda_{ij}^{-}$ is the intensity of a negative interaction, for a node pair {i,j}. In addition, we aim to constrain the latent spaces into polytopes, defining the convex hull of the latent representations and enabling archetypal characterization. For that, we extend the relational AA formulation in two latent spaces to account for independent archetype extraction in these two latent spaces, each responsible for expressing latent similarity based on positive and negative interactions, respectively. We thus define the Skellam rates as:

(4) $$\lambda_{ij}^{+}=\text{exp}(\gamma_{i}+\gamma_{j}-||A_{\left(+\right)}(z_{i}-z_{j})|{|}_{2})$$

 

(5) $$=\text{exp}(\gamma_{i}+\gamma_{j}-||R_{\left(+\right)}ZC_{\left(+\right)}(z_{i}-z_{j})|{|}_{2}).$$

 

(6) $$\lambda_{ij}^{-}=\text{exp}(\delta_{i}+\delta_{j}-||A_{\left(-\right)}(w_{i}-w_{j})|{|}_{2})$$

**Figure 1.**
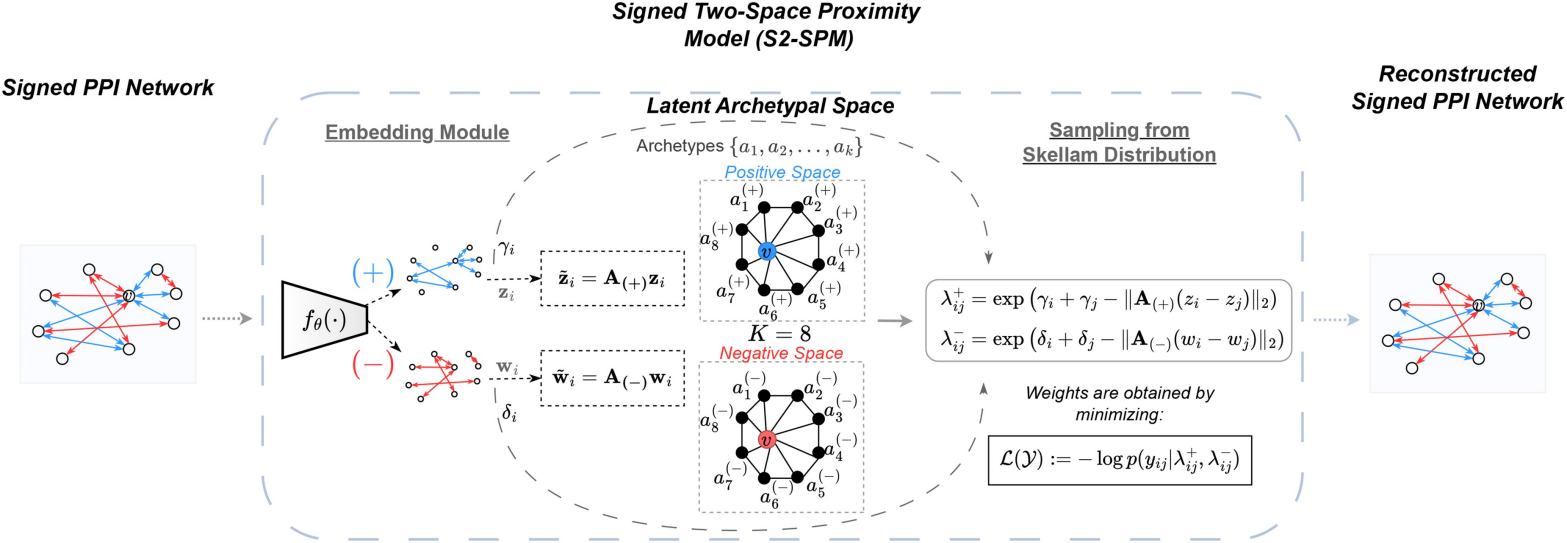
Overview of the proposed Signed Two-Space Proximity Model (S2-SPM). Given a signed protein–protein interaction network as an input, the model assigns two latent vectors $z_{i},w_{i}$ for each of the positive and negative interactions that project each protein to the two archetypal matrices/polytopes $A_{\left(+\right)}$ and $A_{\left(-\right)}$, respectively. Then, embeddings are used to calculate the Skellam rates, optimized for the Skellam log-likelihood, to reconstruct the original signed protein–protein graph

 

(7) $$=\text{exp}(\delta_{i}+\delta_{j}-||R_{\left(-\right)}WC_{\left(-\right)}(w_{i}-w_{j})|{|}_{2}).$$

where $A_{\left(+\right)}$, and $A_{\left(-\right)}$ are the matrices with columns containing the archetypes for the positive and negative latent spaces, respectively. This case differs from the classical AA since the data matrices now refer to latent variables. We define the data matrices as $X_{\left(+\right)}=R_{\left(+\right)}Z$, and $X_{\left(-\right)}=R_{\left(-\right)}W$ with $R_{\left(+\right)},R_{\left(-\right)}\in R^{K\times K}$. Lastly, to guarantee that the archetypes are points belonging to the latent embeddings, we adopt for $C_{\left(+\right)},C_{\left(-\right)}\in R^{N\times K}$ a gated version (Nakis *et al.* 2023) as:

$$\begin{array}{c} c_{(+)nd}=\frac{{\left(Z^{⊤}{}^{\circ}{\left[\sigma (G_{\left(+\right)})\right]}^{⊤}\right)}_{nd}}{\sum_{n^{\prime }}{\left(Z^{⊤}{}^{\circ}{\left[\sigma (G_{\left(+\right)})\right]}^{⊤}\right)}_{n^{\prime }d}},\\ c_{(-)nd}=\frac{{\left(W^{⊤}{}^{\circ}{\left[\sigma (G_{\left(-\right)})\right]}^{⊤}\right)}_{nd}}{\sum_{n^{\prime }}{\left(W^{⊤}{}^{\circ}{\left[\sigma (G_{\left(-\right)})\right]}^{⊤}\right)}_{n^{\prime }d}}, \end{array}$$

with σ(·) the logistic sigmoid function. Finally, ${\left\{\gamma_{i},\delta_{i}\right\}}_{i\in V}$ denote the node-specific random effect terms, and $||\cdot |{|}_{2}$ is the Euclidean distance function. Essentially, $\gamma_{i},\gamma_{j}$ represent the tendency of a node to form positive connections while $\delta_{i},\delta_{j}$ the tendency to form negative connections. In other words, it accounts for degree heterogeneity in the positive and negative sub-networks accordingly. An overview of the proposed method is provided in Fig. 1.

The most closely related work to our method, SLIM (Nakis *et al.* 2023), employs a latent distance-based approach to extract a unified embedding space using a Skellam likelihood. However, SLIM does not distinguish between the two latent spaces and fails to model negative interactions as close proximity, a crucial feature for SPPIs. Similarly, recent work (Nakis *et al.* 2024) introduced two latent membership vectors for positive and negative links, but still projects the embeddings onto a shared latent space. In contrast, our method generalizes this approach by decoupling the positive and negative link characterizations into two completely independent polytopes, one for each interaction type.

#### 2.1.3 Structure retrieval and consistency of archetypes

Our proposed approach defines two latent spaces, which are designed to extract archetypes based on the positive link and negative link structures, respectively. *Protein–protein* networks are known to contain noise and spurious protein interactions (Hashemifar *et al.* 2018; Li et al. 2007; Rao et al. 2014). Consequently, we must verify our models’ ability to extract informative, consistent, and robust structures for such networks. For that, we make use of the Bayesian Normalized Mutual Information (BNMI) metric (Hinrich *et al.* 2016), as shown in Eq. (8). This is a direct consequence of our model defining soft memberships over the archetypes, and thus, classical clustering quality metrics that are defined over (sole) hard memberships are not optimal. Specifically, we follow (Hinrich *et al.* 2016), and we run our model five times (r=5) for each latent dimension *K* of the model. We then concatenate the obtained positive and negative archetypes membership matrices $Z=[Z^{\left(1\right)};Z^{\left(2\right)};Z^{\left(3\right)};Z^{\left(4\right)};Z^{\left(5\right)}]$ and $W=[W^{\left(1\right)};W^{\left(2\right)};W^{\left(3\right)};W^{\left(4\right)};W^{\left(5\right)}]$, respectively. For each case and specification, we calculate the all-pairs BNMI table across the five runs and report the average score and standard deviation based on:

(8) $$\mathrm{BNMI}(Q^{r},Q^{r^{\prime }})=\frac{2I(Q^{r},Q^{r^{\prime }})}{I(Q^{r},Q^{r})+I(Q^{r^{\prime }},Q^{r^{\prime }})},$$

with $I(Z_{\left(+\right)}^{r},Z_{\left(+\right)}^{r^{\prime }})=\sum_{k,k^{\prime }}p(k,k^{\prime })\;\text{log}\;\frac{p(k,k^{\prime })}{p(k)p(k^{\prime })}$, $p(k,k^{\prime })=\sum_{n}p(k/n)p(k^{\prime }/n)(p(n))$ the joint distribution with $p(k/n)=q_{kn}^{r}$ while p(n)=1/N, and $Q^{r}=\{Z^{r}\}$ or $Q^{r}=\{W^{r}\}$. We here note that BNMI is equal to 1 when $Q^{r}=Q^{r^{\prime }}$, and equal to 0 in the case that no common structure/information exists between the two solutions. We also account for structure retrieval sharing due to randomness and calculate the BNMI while randomly permuting the columns of the obtained positive and negative space obtained memberships $Z^{r}$, and $W^{r}$ for each run *r*. Permuting the allocation matrix is expected to destroy the structure, and any shared information will be due to randomness. This procedure allows us to compare the results of our proposed method with the signal one would expect from random noise, providing a metric for their validity.

### 2.2 Experimental setting and evaluation protocols

We continue by providing the experimental setup and setting, the considered datasets and baselines for evaluating the performance and robustness of our proposed framework.

#### 2.2.1 Datasets

We evaluate the proposed method on *protein–protein* interaction networks from the SIGnaling Network Open Resource 3.0 (SIGNOR) (Lo. Surdo et al. 2022). Following this line of work, we construct three signed *protein–protein* interaction networks describing three different organisms; (i) *Homo sapiens*, (ii) *Mus musculus*, and (iii) *Rattus norvegicus*. To infer these networks, we extract all *protein–protein* pairs in the dataset and assign a positive (+) link if the effect describes *up-regulation* and a negative (−) link for *down-regulation*. Finally, we extract the largest connected component for each network. Statistics on the three derived networks are provided in the supplementary.

#### 2.2.2 Baselines

We compare the performance of our model to various state-of-the-art baselines for modeling signed networks. Specifically, (i) SLIM (Nakis *et al.* 2023) is a latent distance model that learns a single embedding matrix optimizing the Skellam likelihood, (ii) POLE (Huang *et al.* 2022) learns the network embeddings by decomposing the signed random walks auto-covariance similarity matrix, (iii) SLF (Xu *et al.* 2019) extracts representations as the concatenation of two latent factors targeting positive and negative relations, (iv) SiGAT (Huang *et al.* 2019) is a graph neural network approach that uses graph attention to update the node embeddings, (v) SDGNN (Huang *et al.* 2021) combines status and balance theory with a graph neural network to reconstruct link signs, link directions, and signed directed triangles via the node embeddings, and (vi) SPMF (Xu *et al.* 2023) uses a low-rank matrix approximation to encode the multi-order signed proximity over a signed network yielding expressive node representations.

#### 2.2.3 Protein Gene Ontology terms

For the proteins included in the datasets, we use the UniProt (Coudert *et al.* 2023) database to extract the Gene Ontology (GO) terms associated with them. These terms belong to three general categories, including (i) *Biological Processes*, (ii) *Molecular Functions*, and (iii) *Cellular Components*. Biological Process refers to the biological objectives to which the gene or gene product contributes, Molecular Function describes the elemental activities of a gene product at the molecular level, such as binding or catalysis, and Cellular Component denotes the parts of a cell or its extracellular environment where the gene product is active. These annotations are used extensively in the biological sciences for various purposes, including interpreting gene expression patterns and protein–protein interactions. The structured vocabulary allows researchers to make meaningful inferences about protein function based on their GO annotations. In our study, we focus on analyzing how specific GO terms are represented in the different interaction types—positive and negative—within our networks. By integrating GO annotations, we can attribute functional characteristics to clusters of proteins that frequently interact either positively or negatively, potentially identifying biological pathways or processes that are predominantly regulated by these interaction types.

#### 2.2.4 Enrichment analysis of archetypes

Here, we continue with the enrichment analysis of the obtained archetypes from the proposed S2-SPM. We consider the model specification defining eight archetypes (K=8) since it provides BNMI≈0.8 for all three datasets, but the analysis can easily be extended to additional dimensions. In addition, the dimensionality of the two spaces, as introduced by S2-SPM, is not required to be the same, that is $K_{\left(+\right)}\neq K_{\left(-\right)}$, we consider though, the case where $K_{\left(+\right)}=K_{\left(-\right)}=K$, for simplicity. Our enrichment analysis, is based on the protein GO terms while we follow a similar strategy as in (Hart *et al.* 2015), to verify the statistical significance and validity of such an analysis. To take into account a particular *GO* term, at least 20 proteins need to be related to that term. For the enrichment analysis of archetype *k*, we start by calculating and sorting the latent distance between every node in the network and the specific archetype. We then define a total of *B* bins, such that each bin contains an equal amount of network nodes, sorted by distance in increasing order. Consequently, the first bin contains the nodes that reside closest to the archetype, while the last bin contains the points that reside furthest from the archetype. We then search for the GO terms/labels that are maximally enriched in the bin closest to the archetype (Hart *et al.* 2015). We define the enrichment value at a bin *b* and for a GO term *l* as $E_{bl}=\rho_{bl}/P_{l}$, where $\rho_{bl}$ is the density of GO term *l* in the bin *b*, and $P_{l}$ the density of GO term *l* in the whole dataset. As in (Hart *et al.* 2015), we compute the significance of the enrichment value in the bin closest to the archetype $E_{b_{0}l}$) via a hypergeometric test. Consequently, each GO term is associated with a *P*-value, describing the significance of the enrichment in the first bin. We consider GO terms with a *P*-value <0.002 as potential candidates for being enriched in the archetype. Furthermore, to account for the false discovery rate (FDR), given by the high number of performed enrichment significance tests, we perform a multiple-hypothesis test using the Benjamini–Hochberg (BH) procedure, setting the FDR level a=0.05. Lastly, for each GO term that is significant under the hypergeometric test (*P*-value <0.002) and survives the FDR procedure, we calculate the probability $p_{\text{max}}$ (Hart *et al.* 2015) that the enrichment value in the first bin $E_{b_{0}l}$ is maximum with regards to the rest of the bins, $E_{b_{0}l}>E_{bl}\;\text{for}\;b>0$. We finally consider a GO term as enriched in a given archetype if it is associated with a probability $p_{\text{max}}>0.5$.

#### 2.2.5 Bin size calculation

An important aspect of the enrichment analysis adopted in this study is the value of the bin size, which defines the number of points in each bin. In (Hart *et al.* 2015), the authors express the minimum bin size such that randomness does not affect the underlying enrichment signal. We here argue that a unique choice for the bin size may not be optimal, as it can lead to a different number of enriched GO terms. Based on that, we consider multiple bin sizes ranging from 1% to 20% of the network size *N* with a step size equal to 1%. We consider the final enriched labels as the ones that are characterized as significant (based on the analysis described above) in at least half of the considered bin sizes, that is yielding a significance appearance rate $\left\{\mathrm{SAR}=\frac{\#\;\text{bins}\;\text{sizes}\;\text{label}\;\text{is}\;\text{significant}}{\#\;\text{total}\;\text{bin}\;\text{sizes}}\geq 0.5\right\}$. By aggregating results based on multiple bin sizes, we argue that any strong dependencies between the enrichment analysis and the choice of the bin size value are removed.

## 3 Results

Next, we evaluate the representation capabilities of the proposed model from quantitative and qualitative aspects. Specifically, we assess its effectiveness in link prediction and in successfully inferring positive and negative archetypes and communities, accompanied by enrichment analysis.

### 3.1 Robustness and identifiability of the solution

**Figure 2.**
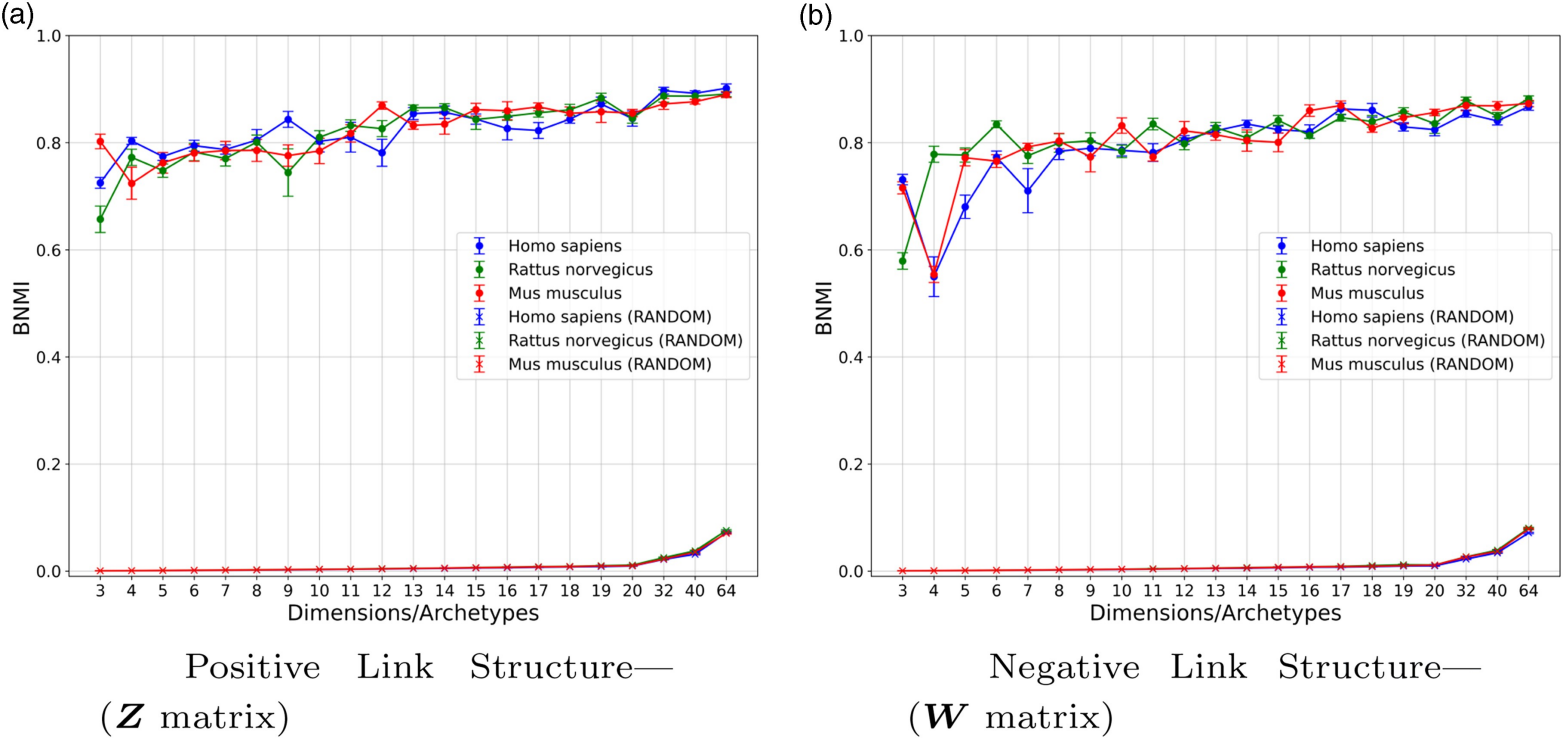
BNMI: Robustness of solution and structure characterization of S2-SPM, as a function of the number of dimensions/archetypes across five reruns, and three networks. For a given dataset, the (RANDOM) labeled lines denote the BNMI value of the solution that should be expected by luck, for a given choice for the number of archetypes/dimensions.

An important aspect of a model characterizing the structure of a given signed network is the robustness, as well as the identifiability of the given solution. For that, we here present the results of the BNMI across five model reruns, as described in the previous section. Specifically, in Fig. 2a and b, we provide the BNMI scores for both the positive and negative space mixed-membership matrices Z,W, respectively. Essentially, we compare the consistency of the soft assignments with respect to the archetypes across the different runs of the model. We also provide the BNMI scores that should be expected by chance, presented with (RANDOM) in the legends of the aforementioned figures. We consider all three datasets for various dimensions, ranging from 3 to 64. For the positive space mixed-membership matrix Z, we observe that the BNMI has a gradual and modest increase as the number of dimensions grows for all three datasets. For the (RANDOM) permutations, the BNMI scores are essentially zero for all dimensions of less than 32, and show very small values for greater dimensions. For the negative space mixed-membership matrix W, we notice the same behavior as in the positive case, with the main difference that in model configurations with fewer than 7 archetypes, the BNMI score fluctuates more. Based on these results, we conclude that the model obtains consistent results and high BNMI scores across reruns when K>4 for the positive case and K>6 for the negative case. Thus, our S2-SPM yields successful and robust structure characterization. In the case of the randomly permuted solutions, the BNMI scores are 0, validating that there is no structure retrieval due to randomness. To calculate the BNMI score that should be expected by luck, we have considered 100 permutations of the solution for every, reporting the average scores across reruns.

### 3.2 S2-SPM significantly outperforms baselines in signed link prediction

To evaluate the predictive capability of our model, we consider the general task of signed link prediction. Contrary to previous works (Nakis *et al.* 2023; Xu *et al.* 2019; Huang *et al.* 2022), we extend the experimental setting beyond binarized sign prediction and link prediction. We instead formulate the task as a three-class classification problem [negative (*neg*), zero (*zr*), and positive (*pos*) links]. For this task, we remove (or set to zero) 10% of the total network links while ensuring that the remaining network stays connected. We then train the model on this residual network and evaluate its ability to simultaneously predict both the sign and the existence of the removed links. Specifically, for a given pair of proteins, our goal is to determine whether they exhibit negative regulation (*neg*), positive regulation (*pos*), or no interaction at all (*zr*). (Additional settings are provided in the supplementary.)

We compare our models’ performance against seven prominent methods for signed networks. We present results regarding per-class and total weighted (*w*) F1 scores in Table 1. We here witness that S2-SPM outperforms, and in most cases significantly, all the considered baselines in all classes and on the weighted F1 score across classes. Table 1 further shows that the negative regulation class (*neg*) is the most challenging to predict, highlighting the importance of models that account for this setting. Regarding the baselines, SLIM is the most competitive method following S2-SPM, highlighting the superiority of latent distance modeling in link prediction. Importantly, only the latent distance models achieved high scores in all classes of the considered problem, with SLF also having favorable performance. The rest of the baselines were observed to be competitive only to a subset of the tasks and thus can be characterized as non-robust in their predictive performance. Overall, the proposed S2-SPM demonstrated improved predictive performance for all three datasets.

**Table 1.** Per class (*pos*, *zr*, *neg*) and weighted (*w*) F1-scores for representation size K=8.

	*Homo sapiens*				*Mus musculus*				*Rattus norvegicus*			
Class	*neg*	*zr*	*pos*	*w*	*neg*	*zr*	*pos*	*w*	*neg*	*zr*	*pos*	*w*
POLE	0.292	0.738	0.413	0.563	0.317	0.746	0.449	0.585	0.373	0.758	0.465	0.603
SLF	0.525	0.832	0.683	0.740	0.526	0.826	0.661	0.729	0.504	0.823	0.670	0.729
SiGAT	0.278	0.691	0.513	0.575	0.322	0.679	0.503	0.572	0.375	0.713	0.563	0.618
SDGNN	0.447	0.735	0.565	0.637	0.459	0.729	0.557	0.633	0.435	0.721	0.564	0.629
SPMF	0.375	0.689	0.558	0.602	0.344	0.680	0.549	0.593	0.318	0.687	0.582	0.606
SLIM	0.463	0.826	0.649	0.716	0.445	0.822	0.652	0.715	**0.506**	0.836	0.682	0.740
S2-SPM	**0.562**	**0.852**	**0.704**	**0.761**	**0.541**	**0.851** ^a^	**0.702**	**0.759**	0.504	**0.863**	**0.706**	**0.763**

a Bold indicates the best-performing model, while underline marks the second-best performer.

### 3.3 Enrichment analysis of the archetypes

To validate the biological relevance of the identified archetypes, we next analyze the GO terms that were found to be enriched in each archetype, focusing on the *Homo sapiens* network.

#### 3.3.1 Down-regulation archetypes enrichment analysis

We found some identified archetypes to be enriched for proteins associated with distinct biological processes. Among these, archetype 5 represents the processes of antiviral immune response: defence response to viruses, innate immune response, mRNA binding, positive regulation of interferon-alpha production, and positive regulation of type I interferon production. Specifically, it captures positive regulation of interferon-alpha, a type I interferon predominantly produced by innate immune cells in response to viral infection (Ivashkiv and Donlin 2014; Katze *et al.* 2002). Similarly, archetype 8 captures key processes and components with common biological relevance integral to mitosis, including cell division, structural constituents of the cytoskeleton, mitotic cell cycle, mitotic spindle, microtubule cytoskeleton, microtubule, microtubule cytoskeleton organization, myosin phosphatase activity, and MAPK cascade (Walczak *et al.* 2008). Collectively, this archetype captures the last phase of the cell cycle, a type of cell division essential for growth, development, and repair (Maiato, 2021; Zhang and Liu 2002). Archetype 4 is primarily involved in regulating Rho GTPase signaling, which is crucial in various biological processes, such as cell cytoskeletal organisation, differentiation, growth, neuronal development, and synaptic functions (Moon & Zheng 2003). Other archetypes were more complex to interpret as they reflect multiple biological processes. Archetype 1 is centered around cell migration, adhesion, and tissue organization, in the context of development, epithelial to mesenchymal transition (EMT), and extracellular matrix remodeling. Enrichment in the transforming growth factor (TGF)-β signaling pathway, essential for EMT and migration, both known for their importance during development and cancer progression (Takahashi *et al.* 2022; Massagué, 2008). Archetype 2 is mainly associated with G protein-coupled receptor (GPCR) signaling, synaptic transmission, and cell signaling at the plasma membrane. Its enrichment in key roles in calcium signaling, neuropeptide signaling, and cell adhesion indicates its involvement in modulating neuronal communication and cellular interactions. Archetype 3, features its key role in cell-cell signaling, processes related to the extracellular space and matrix, and hormonal and ligand-receptor activities. Archetype 6 is the smallest among the archetypes and seems to represent a dual function: one in mitochondrial protein synthesis and the other in chromatin structure. Archetype 7 integrates processes related to autophagy, apoptosis, and cell division. It likely reflects the role of mitophagy, determining whether a cell under mitochondrial stress survives through repair (autophagy) or undergoes cell death (apoptosis) (Chiara Maiuri et al. 2007).

#### 3.3.2 Up-regulation archetypes enrichment analysis

All positively regulated archetypes are relatively large, often encompassing diverse GO terms, which contribute to their complexity and the challenge of assigning a specific biological function, while also implying dynamic interactions among their components. Archetype 2 stands out with a direct biological association, as it is predominantly linked to the cell cycle, with a strong emphasis on cytoskeletal organization, chromosome segregation, and DNA repair processes. Both archetypes 4 and 7 are primarily associated with protein homeostasis, with a strong emphasis on regulating protein degradation via ubiquitination (Tai and Schuman 2008; Sahu *et al.* 2023). These seem to reflect the two major pathways for protein degradation: autophagy (archetype 4) and ubiquitin–proteasome system (archetype 7) (Lilienbaum, 2013). Archetype 5 is strongly associated with G protein-coupled protein activity and regulation, neurotransmission, hormone activity, and calcium signaling, implying its role in synaptic signaling (Gerber *et al.* 2016). Archetype 8 is the largest and encompasses both innate and adaptive immune responses and inflammation. The rest of the archetypes exhibit mixed biological signals with dynamic interplay, including bone development and differentiation, TGF-β signaling, morphogenesis, transcription, and immune response regulation (archetype 1); translation, cell cycle regulation, synaptic transmission, and neural activity (archetype 3); multiple processes, among which chromatin organization and Wnt signaling (archetype 6).

In summary, the identified positive and negative space archetypes are often complex but reflect significant biological processes. This complexity is likely influenced by protein interaction signals captured across various cell types, each differentiated by the nature and levels of specific proteins they express. As UniProt integrates data from both healthy and diseased sources, it may already capture dysregulation in its proteome due to pathological conditions or has the potential to detect it (examples of the enrichment labels are provided in the supplementary).

#### 3.3.3 PPI network visualizations

**Figure 3.**
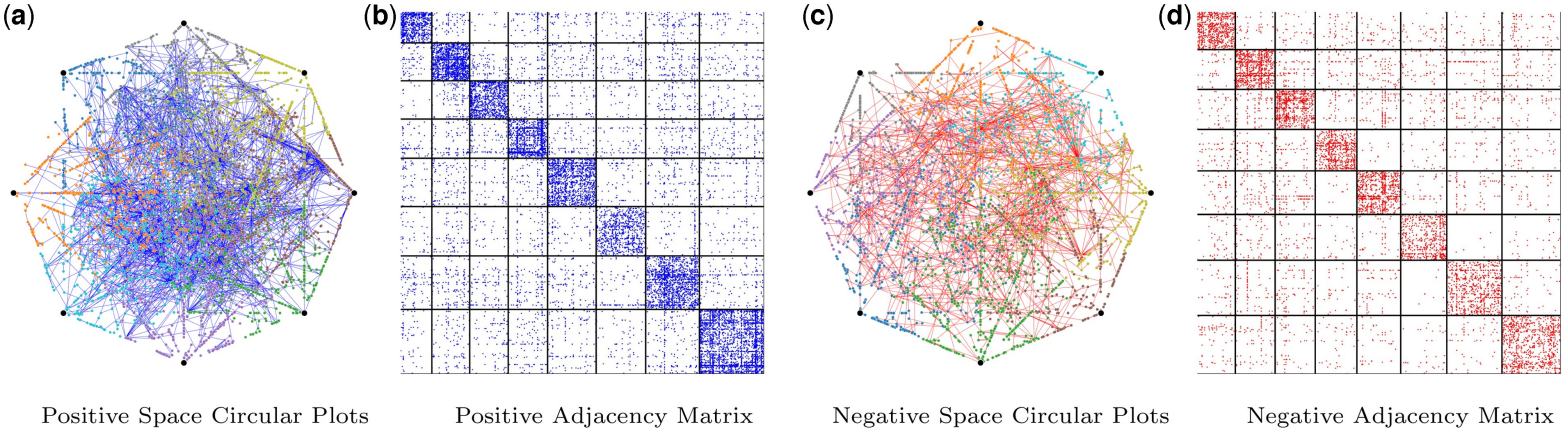
*Homo sapiensS2-SPM (K = 8)*: Positive space (a) and (b), and negative space (c) and (d) inferred simplex visualizations and ordered adjacency matrices for K=8 archetypes. (a) and (c), provide the Positive/Negative Space Circular Plot (PSCP)/(NSCP) with blue/red lines showcasing positive/negative edges between proteins—Figure 3 (b) and (d), show the Ordered Positive/Negative Edges Adjacency (OrA) matrices sorted based on the memberships $z_{i}$/$w_{i}$, in terms of maximum simplex corner responsibility.

We present visualizations obtained from the proposed S2-SPM, demonstrating its capability to extract informative and robust latent structures. Figure 3 illustrates the inferred latent structures for both positive and negative spaces as defined by the model. Specifically, Fig. 3a and c, depict the projections of the positive and negative space latent embeddings, $\tilde{Z}=A_{\left(+\right)}Z$ and $\tilde{W}=A_{\left(-\right)}W$, onto circular plots. These are enriched with edges connecting nodes assigned to the same archetype. Each archetype is represented as a point evenly spaced around a circle, positioned every ${\text{rad}}_{k}=\frac{2\pi }{K}$ radians, where *K* is the total number of archetypes. The circular plots highlight S2-SPM ’s ability to allocate nodes to distinct archetypes, revealing proteins behaving as archetypes or extreme profiles within the data. Figure 3b and d display the reordered adjacency matrices based on the archetype assignments of the positive and negative mixed-membership matrices Z and W. These matrices effectively uncover the underlying latent or block structures in the data. This visualization demonstrates S2-SPM ’s effectiveness in identifying and characterizing the archetypes and latent structures inherent in the data, offering insights into the underlying biological processes.

## 4 Discussion

Automatically predicting interactions in complex biological networks remains a challenging yet crucial step for solving several biological tasks, including decoding disease mechanisms and accurately determining therapeutic targets. In this study, we proposed the Signed Two-Space Proximity Model (S2-SPM), tailored to the machine learning modeling of signed PPI networks. Specifically, we introduced two latent spaces to decouple positive and negative network interactions, assuming that both interaction types should be translated into close proximity in a latent space model. Prominent modeling techniques for PPI networks are typically blind to the sign of interactions, a limitation addressed by the proposed model.

Additionally, our method addresses the circularity concerns that commonly arise in classical archetypal analysis studies (Hart *et al.* 2015), where the same data are used both to define the archetypes and to identify the enriched labels or traits within each archetype. In contrast, S2-SPM leverages the signed PPI network to identify the archetypal structure. This process is independent of each protein’s underlying labels or GO terms. These labels are only used later for enrichment analysis. As a result, the two stages are decoupled, eliminating the risk of data leakage and the need for additional validation steps (Hart *et al.* 2015). This design choice further highlights the superiority of our method.

In experiments, S2-SPM outperformed all baselines, particularly in F1 scores for the signed link prediction task. Notably, our model significantly surpassed recent signed network models based on latent distance and the Skellam distribution (Nakis *et al.* 2023), emphasizing the importance of using two independent latent spaces for modeling each interaction type in signed protein–protein networks. Furthermore, we presented that the obtained archetype structures could further be enriched with the GO terms characterizing the different proteins present in the *homo-sapiens* network. Specifically, we showcased that both positive and negative interactions formed archetypal groups carrying out different biological tasks. The obtained archetype structures were also tested for statistical significance, robustness, and spurious structure retrieval. Comparisons with information signals given by chance under random permutations of the archetypal membership matrix also confirmed that our model yields reliable and consistent structures for both latent spaces. This analysis proves that S2-SPM constitutes an identifiable approach for modeling complex protein interactions while ensuring that key biological features are precisely captured and interpretable.

## Supplementary Material

btaf204_Supplementary_Data

## Data Availability

The original data for creating the signed protein–protein interaction networks are publicly available at the SIGnaling Network Open Resource 3.0 portal: https://signor.uniroma2.it/downloads.php. The Gene Ontology (GO) terms for the corresponding proteins are publicly available and can be accessed at the UniProt database: https://www.uniprot.org.
