# Supplementary material for: The signed two-space proximity model for learning representations in protein–protein interaction networks
Source: Bioinformatics. 2025 Apr 23;41(6):btaf204. doi: 10.1093/bioinformatics/btaf204 (PMC12129585; doi:10.1093/bioinformatics/btaf204)
Supplement: btaf204_Supplementary_Data [file btaf204_supplementary_data.pdf]

# Supplementary Material

## Dataset Details

Statistics on the three derived networks are provided in Table 1, including their number of nodes $|\mathcal{V}|$, number of positive $|\mathcal{Y}^+|$ and negative edges $|\mathcal{Y}^-|$, as well as their density.

**Table 1.** Protein-Protein Interaction network statistics; $|\mathcal{V}|$: # Nodes, $|\mathcal{Y}^+|$: # Positive links, $|\mathcal{Y}^-|$: # Negative links.

| | $\|\mathcal{V}\|$ | $\|\mathcal{Y}^+\|$ | $\|\mathcal{Y}^-\|$ | Density |
|---|---|---|---|---|
| *Homo sapiens* | 5,645 | 8,665 | 4,768 | 0.0008 |
| *Mus musculus* | 5,242 | 8,119 | 4,512 | 0.0009 |
| *Rattus norvegicus* | 4,914 | 7,539 | 4,229 | 0.0010 |

## Link Prediction

To evaluate the predictive capability of our model, we also follow the experimental set-up of (Nakis et al., 2023; Xu et al., 2019; Huang et al., 2022). We consider two tasks as 1) *Link sign prediction*; where we remove/set to zero 10% of the total network links while keeping the residual network connected. We train the model based on the residual network, and then we test the model on its ability to predict the sign of the removed links during inference. 2) *Signed link prediction*; where we follow the same link removal strategy but we increase the difficulty of the previous task since now the model has to predict both the existence and sign of the removed link during inference. To construct the test set, we combine the removed links with a sample of the same number of node pairs that are not connected by edges in the original network to create zero instances. Finally, to account for the high link/non-link imbalance being present in the data we adopt the area-under-curve of the receiver operating characteristic (AUC-ROC) and precision-recall (AUC-PR) curves. For our method we follow (Nakis et al., 2023), and fit a logistic regression classifier on the concatenation of the corresponding Skellam rates and log-rates, as $\chi_{ij} = \left[\lambda_{ij}^+, \lambda_{ij}^-, \log\lambda_{ij}^+, \log\lambda_{ij}^-\right]$. Such an action utilizes both a linear function of the rates and their ratio or product via the log transformation. To generate feature vectors for the baselines, we employ five binary operators average, weighted L1, weighted L2, concatenate, Hadamard product. For each feature vector, we train a logistic regression model except for the Hadamard product, which can be used directly for predictions. Given that each operator yields varying performances, we select the operator that yields the highest performance for each individual task when constructing the baselines, as in (Nakis et al., 2023). We denote the task of the link sign prediction task as $p@n$. The tasks of signed link prediction between positive and zero samples are denoted as $p@z$, while the negative against zero is denoted as $n@z$.

**Table 2.** Area Under Curve (AUC-ROC) scores for representation size of $K = 8$.

| | *Homo sapiens* | | | *Mus musculus* | | | *Rattus norvegicus* | | |
|---|---|---|---|---|---|---|---|---|---|
| Task | $p@n$ | $p@z$ | $n@z$ | $p@n$ | $p@z$ | $n@z$ | $p@n$ | $p@z$ | $n@z$ |
| POLE | .756 | .737 | .640 | .781 | .717 | .703 | .753 | .707 | .655 |
| SLF | .842 | .892 | .954 | .838 | .888 | .961 | .838 | .891 | .957 |
| SIGAT | .753 | .637 | .658 | .790 | .640 | .752 | .772 | .651 | .725 |
| SDGNN | .813 | .683 | .743 | .839 | .674 | .825 | .811 | .718 | .829 |
| SPMF | .838 | .819 | .902 | .836 | .811 | .904 | .810 | .799 | .917 |
| SLIM | .827 | .897 | .933 | .837 | **.902** | .947 | .808 | .895 | .941 |
| S2-SPM (OURS) | **.863** | **.898** | **.969** | **.867** | .898 | **.965** | **.839** | **.903** | **.972** |

**Table 3.** Area Under Curve (AUC-PR) scores for representation size of $K = 8$.

| | *Homo sapiens* | | | *Mus musculus* | | | *Rattus norvegicus* | | |
|---|---|---|---|---|---|---|---|---|---|
| Task | $p@n$ | $p@z$ | $n@z$ | $p@n$ | $p@z$ | $n@z$ | $p@n$ | $p@z$ | $n@z$ |
| POLE | .894 | .775 | .518 | .918 | .769 | .586 | .907 | .761 | .577 |
| SLF | .946 | .868 | .831 | .949 | .874 | .851 | .947 | .869 | .838 |
| SIGAT | .901 | .615 | .338 | .928 | .607 | .400 | .922 | .615 | .349 |
| SDGNN | .938 | .544 | .319 | .952 | .608 | .443 | .942 | .653 | .547 |
| SPMF | .944 | .768 | .692 | .950 | .772 | .687 | .939 | .742 | .724 |
| SLIM | .936 | .889 | .831 | .947 | .894 | .824 | .936 | .887 | .847 |
| S2-SPM (OURS) | **.954** | **.895** | **.895** | **.960** | **.898** | **.873** | **.950** | **.904** | **.900** |

## Training details

We continue by providing the experimental set-up, the considered datasets and baselines for evaluating the performance and robustness of our proposed framework. All experiments regarding our proposed model and baselines have been conducted on an 8 GB NVIDIA RTX 2070 Super GPU. Furthermore, for training, we used the Adam optimizer (Kingma and Ba, 2017) with a fixed learning rate $\mathrm{lr} = 0.05$ and performed 5000 iterations. Lastly, the proposed S2-SPM was initialized based on the furthest-sum algorithm (Mørup et al., 2010; Nakis et al., 2023).

## Additional Network Visualizations

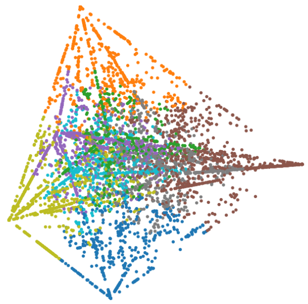

(a) PCA—first two Principal Components

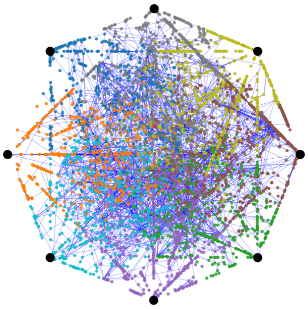

(b) Positive Space Circular Plots

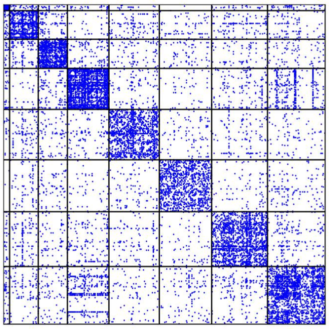

(c) Ordered Positive Adjacency Matrix

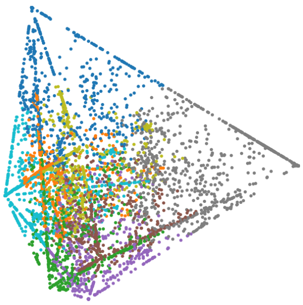

(d) PCA—first two Principal Components

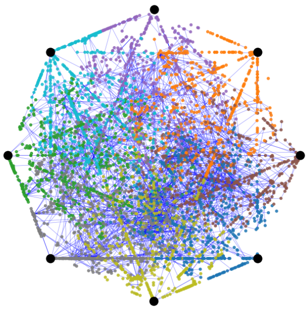

(e) Positive Space Circular Plots

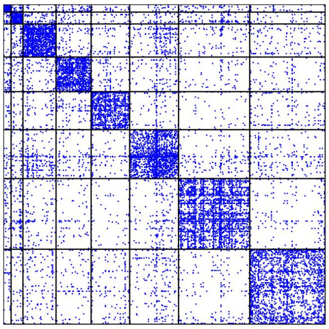

(f) Ordered Positive Adjacency Matrix

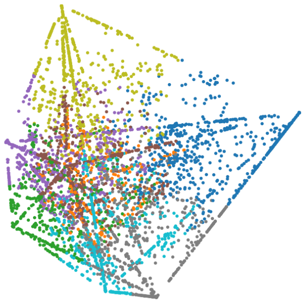

(g) PCA—first two Principal Components

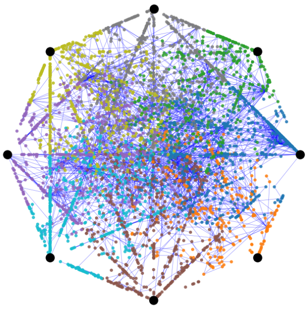

(h) Positive Space Circular Plots

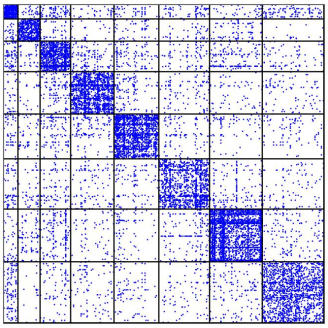

(i) Ordered Positive Adjacency Matrix

**Fig. 1. S2-SPM(K=8)**: Multiple Networks—Positive space inferred simplex visualizations and ordered adjacency matrices for $K = 8$ archetypes. The first column shows the latent space projection to the first two Principal Components of the positive latent space $\boldsymbol{Z}$—The second column provides the Positive Space Circular Plot (PSCP) with blue lines showcasing positive edges between proteins—The third columns shows the Ordered Positive Edges Adjacency (OrA) matrices sorted based on the memberships $\mathbf{z}_i$, in terms of maximum simplex corner responsibility, and internally according to the magnitude of the corresponding corner assignment for their reconstruction. Each row describes a different dataset, *Homo sapiens*, *Mus musculus*, and *Rattus norvegicus*, respectively.

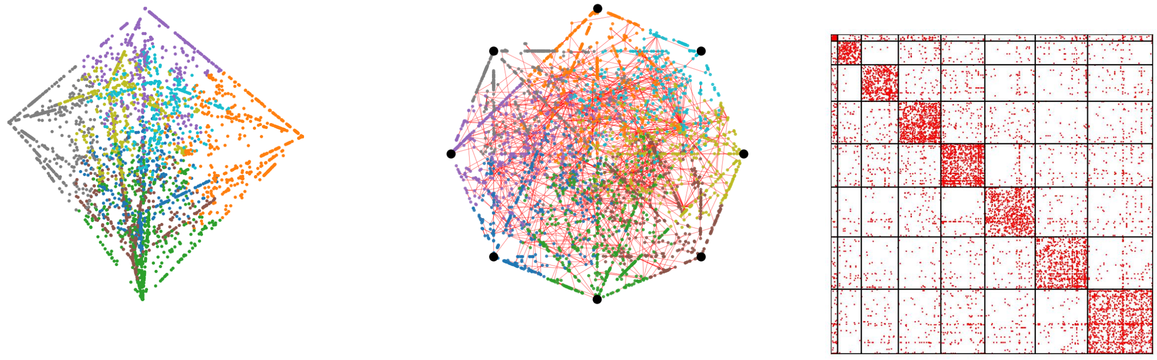

(a) PCA—first two Principal Components (b) Negative Space Circular Plots (c) Ordered Negative Adjacency Matrix

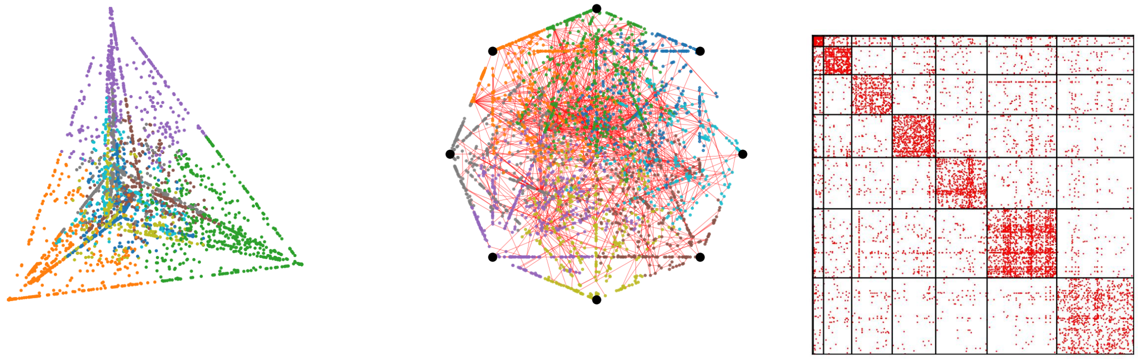

(d) PCA—first two Principal Components (e) Negative Space Circular Plots (f) Ordered Negative Adjacency Matrix

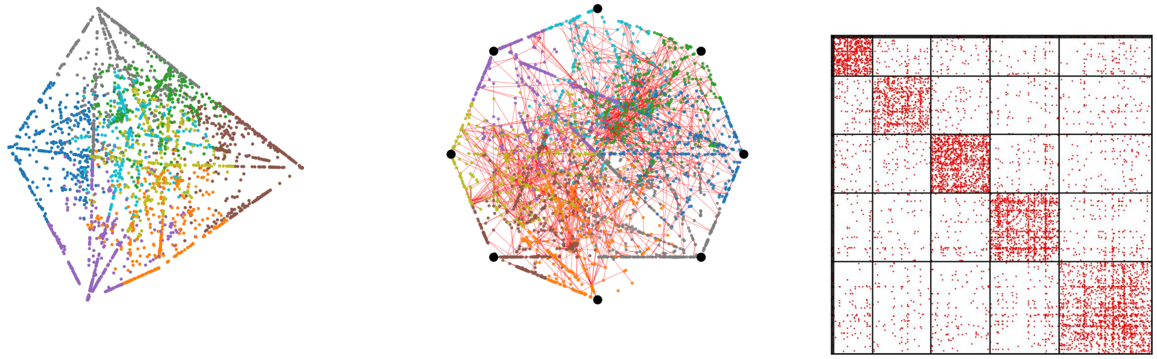

(g) PCA—first two Principal Components (h) Negative Space Circular Plots (i) Ordered Negative Adjacency Matrix

**Fig. 2. S2-SPM(K=8)**: Multiple Networks—Negative space inferred simplex visualizations and ordered adjacency matrices for $K = 8$ archetypes. The first column shows the latent space projection to the first two Principal Components of the negative latent space $\boldsymbol{W}$—The second column provides the Negative Space Circular Plot (NSCP) with red lines showcasing negative edges between proteins—The third columns shows the Ordered Negative Edges Adjacency (OrA) matrices sorted based on the memberships $\mathbf{w}_i$, in terms of maximum simplex corner responsibility, and internally according to the magnitude of the corresponding corner assignment for their reconstruction. Each row describes a different dataset, *Homo sapiens*, *Mus musculus*, and *Rattus norvegicus*, respectively.

## Examples of enriched GO terms at each archetype

**Table 4.** *Homo sapiens*—Negative space archetype enrichment analysis for representation size of $K = 8$.

| Archetype 1 | Archetype 2 | Archetype 3 | Archetype 4 |
|---|---|---|---|
| *P:positive regulation of cell migration*<br>*P:cell morphogenesis*<br>*P:positive regulation of epithelial to mesenchymal transition*<br>*P:extracellular matrix organization*<br>*P:transforming growth factor beta receptor signaling pathway* | *P:G protein-coupled receptor signaling pathway*<br>*P:positive regulation of cytosolic calcium ion concentration*<br>*P:chemical synaptic transmission*<br>*P:cell surface receptor signaling pathway*<br>*P:cell adhesion* | *F:guanyl-nucleotide exchange factor activity*<br>*F:hormone activity*<br>*P:cell-cell signaling*<br>*F:receptor ligand activity*<br>*C:extracellular space* | *F:GTPase activator activity*<br>*P:regulation of small GTPase mediated signal transduction*<br>*P:Rho protein signal transduction*<br>*C:late endosome membrane* |
| **Archetype 5** | **Archetype 6** | **Archetype 7** | **Archetype 8** |
| *P:defense response to virus*<br>*P:innate immune response*<br>*F:mRNA binding*<br>*P:positive regulation of interferon-alpha production*<br>*P:positive regulation of type I interferon production* | *C:mitochondrial matrix*<br>*F:tRNA binding*<br>*C:nucleosome* | *C:mitochondrion*<br>*P:positive regulation of autophagy*<br>*P:intrinsic apoptotic signaling pathway in response to DNA damage*<br>*P:positive regulation of intrinsic apoptotic signaling pathway* | *P:cell division*<br>*P:mitotic cell cycle*<br>*P:MAPK cascade*<br>*P:microtubule cytoskeleton organization*<br>*F:myosin phosphatase activity* |

**Table 5.** *Homo sapiens*—Positive space archetype enrichment analysis for representation size of $K = 8$.

| Archetype 1 | Archetype 2 | Archetype 3 | Archetype 4 |
|---|---|---|---|
| *P:osteoblast differentiation*<br>*P:transforming growth factor beta receptor signaling pathway*<br>*P:cell morphogenesis*<br>*P:positive regulation of immune response*<br>*P:regulation of transcription by RNA polymerase II* | *P:microtubule cytoskeleton organization*<br>*P:chromosome segregation*<br>*C:chromosome, centromeric region*<br>*P:DNA repair* | *P:translation*<br>*P:mitotic cell cycle*<br>*P:modulation of chemical synaptic transmission* | *P:ubiquitin-dependent protein catabolic process*<br>*P:macroautophagy*<br>*P:protein ubiquitination* |
| **Archetype 5** | **Archetype 6** | **Archetype 7** | **Archetype 8** |
| *P:G protein-coupled receptor signaling pathway*<br>*P:cellular response to hormone stimulus*<br>*P:neuropeptide signaling pathway*<br>*P:calcium-mediated signaling* | *F:structural constituent of chromatin*<br>*P:Wnt signaling pathway* | *P:ubiquitin-dependent protein catabolic process*<br>*P:proteasome-mediated ubiquitin-dependent protein catabolic process* | *P:defense response to virus*<br>*P:defense response to bacterium*<br>*P:inflammatory response*<br>*P:innate immune response*<br>*P:adaptive immune response* |

## References

Z. Huang et al. Pole: Polarized embedding for signed networks. *WSDM*, pages 390–400, 2022.

D. P. Kingma and J. Ba. Adam: A method for stochastic optimization, 2017.

M. Mørup et al. Archetypal analysis for machine learning. In *2010 IEEE International Workshop on Machine Learning for Signal Processing*, pages 172–177, 2010.

N. Nakis et al. Characterizing polarization in social networks using the signed relational latent distance model. In *Proceedings of The 26th International Conference on Artificial Intelligence and Statistics*, volume 206 of *Proceedings of Machine Learning Research*, pages 11489–11505. PMLR, 25–27 Apr 2023.

P. Xu et al. Link prediction with signed latent factors in signed social networks. *Proceedings of the Acm Sigkdd International Conference on Knowledge Discovery and Data Mining*, pages 1046–1054, 2019.
